# Movement Behavior of High-Heeled Walking: How Does the Nervous System Control the Ankle Joint during an Unstable Walking Condition?

**DOI:** 10.1371/journal.pone.0037390

**Published:** 2012-05-16

**Authors:** Tine Alkjær, Peter Raffalt, Nicolas C. Petersen, Erik B. Simonsen

**Affiliations:** 1 Department of Neuroscience and Pharmacology, University of Copenhagen, Copenhagen, Denmark; 2 Department of Exercise and Sport Sciences, University of Copenhagen, Copenhagen, Denmark; Universidad Europea de Madrid, Spain

## Abstract

The human locomotor system is flexible and enables humans to move without falling even under less than optimal conditions. Walking with high-heeled shoes constitutes an unstable condition and here we ask how the nervous system controls the ankle joint in this situation? We investigated the movement behavior of high-heeled and barefooted walking in eleven female subjects. The movement variability was quantified by calculation of approximate entropy (ApEn) in the ankle joint angle and the standard deviation (SD) of the stride time intervals. Electromyography (EMG) of the soleus (SO) and tibialis anterior (TA) muscles and the soleus Hoffmann (H-) reflex were measured at 4.0 km/h on a motor driven treadmill to reveal the underlying motor strategies in each walking condition. The ApEn of the ankle joint angle was significantly higher (p<0.01) during high-heeled (0.38±0.08) than during barefooted walking (0.28±0.07). During high-heeled walking, coactivation between the SO and TA muscles increased towards heel strike and the H-reflex was significantly increased in terminal swing by 40% (p<0.01). These observations show that high-heeled walking is characterized by a more complex and less predictable pattern than barefooted walking. Increased coactivation about the ankle joint together with increased excitability of the SO H-reflex in terminal swing phase indicates that the motor strategy was changed during high-heeled walking. Although, the participants were young, healthy and accustomed to high-heeled walking the results demonstrate that that walking on high-heels needs to be controlled differently from barefooted walking. We suggest that the higher variability reflects an adjusted neural strategy of the nervous system to control the ankle joint during high-heeled walking.

## Introduction

The human locomotor system is a flexible system that enables healthy subjects to move without falling even under less than optimal conditions such as in darkness, uneven terrains, on ice or walking with high-heeled shoes. Studies of high-heeled walking have suggested that walking with high-heeled shoes constitutes an unstable walking condition and can be hazardous to balance [1], [2], [3]. The biomechanics and muscle activity are significantly changed during high-heeled walking when compared to normal walking [4], [5], [6], [7], [8], [9], [10], [11]. Increases in the activity in trunk and leg muscles characterizes high-heeled walking [4], [8], [10], [11], [12] as well as an increased metabolic energy cost [13]. This may indicate that high-heeled walking requires a specific neural control different from barefooted walking. Millions of women in Western Societies [14], [15] frequently walk in high heels and typically without falling. Thus, despite the fact that high-heeled shoes may challenge the balance control during walking, the locomotor system seems able to adapt to this condition. Theoretically, the property of the human locomotor control system to cope with less than optimal conditions can be explained by the principle of optimality in movement variability, which recently has been proposed by Stergiou et al. (2006). In this model the movement variability has a deterministic structure reflecting the adaptability of the system to environmental stimuli [16], [17]. Cyclic or repeated movements, such as walking, vary and are never true copies of each other [18]. Thus, variability may be considered a natural and healthy feature in the control of human movement and by quantifying this we get an idea of the behavioral state of the locomotor control system. The variability of cyclic movements may be classified as more (periodic or stereotypic) or less predictable (random) and it is proposed that in a healthy situation an optimal state of movement variability exists, which is characterized by a rather complex and chaotic pattern and placed somewhere in between the purely periodic and random movement pattern [16]. Experimental data suggests that subjects with neuromuscular pathologies or injuries exhibit movement variability with either more predictable (like a robot) [19] or random [20] structures. Approximate entropy (ApEn) is a nonlinear dynamical tool to quantify the complexity of a signal [21]. If a signal contains many repetitive patterns it has a relatively small ApEn while a less predictable (i.e. more complex) signal has a higher ApEn value. ApEn has been applied to understand the behavior of different biological systems and signals [22], [23], [24]. In the present study we investigated the movement behavior of high-heeled walking to explore how the nervous system controls the ankle joint during such an unstable walking condition. We anticipate that walking on high heels is a more complicated task for the nervous system because more degrees of freedom have to be controlled in order to keep balance. This may result in an increased central processing during the control of high-heeled walking, which may increase the movement variability. In support of this, it has recently been suggested that feed forward mechanisms are involved in the control of ankle joint instability [25]. Accordingly, we hypothesized that high-heeled walking would be characterized by a more complex and less predictable behavior than barefooted walking. The movement variability of barefooted and high-heeled walking was quantified by calculation of ApEn in the ankle joint angle and by the standard deviation (SD) of the stride intervals in both walking conditions. To explore the underlying motor strategies in each walking condition, we measured surface electromyography (EMG) of the ankle joint muscles (i.e. the soleus (SO) and tibialis anterior (TA) muscles) and the SO Hoffmann (H-) reflex modulation. During human walking the SO H-reflex has been reported to follow a modulation pattern characterized by facilitation during the stance phase and inhibition during the swing phase [26], [27], [28]. The mechanisms involved in the control of this modulation may be both pre- and postsynaptic but presynaptic inhibition has been suggested to have a substantial influence on the modulation pattern of the SO H-reflex [26], [29], [30]. In addition to this, the muscle activities of the TA and SO muscles have been reported to alternate and thus, limited coactivation between ankle joint agonist and antagonists is present during normal walking [31]. We demonstrate that the ankle joint movement behavior of high-heeled walking is characterized by increased movement variability. We suggest that this reflects the adaptability of the nervous system to cope with the unstable walking condition.

## Results

**Figure 1:**
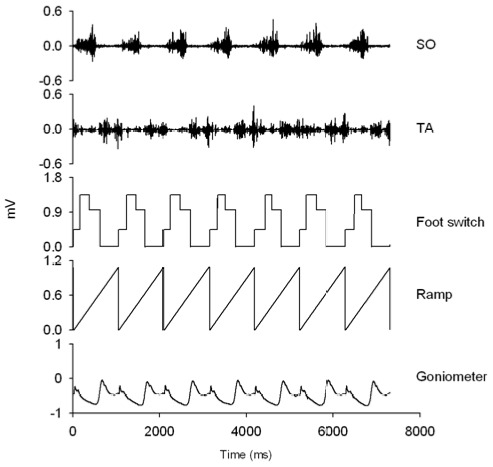
Representative experimental recordings. From top: 1) Raw EMG recordings of SO, TA, 2) foot switch signal, 3) ramp function, which was reset at heel strike, 3) goniometer signal showing the ankle joint angle. All signals are raw and expressed in mV.

**Figure 2:**
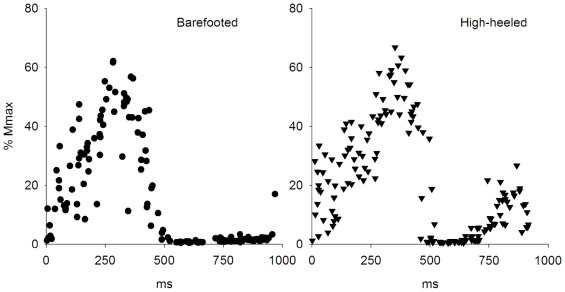
Recordings of SO H-reflex from one subject. Data recordings of SO H-reflex (% Mmax) from one subject during barefooted (dots) and high-heeled (triangles) walking. The stimuli to elicit the H-reflexes were given every 2 s corresponded to 25% (±10%) of the resting Mmax and were slightly out of phase with the gait cycle, which ensured the stimuli to be dispersed randomly over the gait cycle (see text for further explanations).

All subjects walked barefooted and with high-heeled shoes on a motor driven treadmill at 4.0 km/h. Each walking condition consisted of two separate trials. In one trial electromyography (EMG) of the SO and TA muscles, ankle joint kinematics and temporal gait cycle parameters (Figure 1) were collected for 60 s of walking. During another trial the SO H-reflex amplitudes were measured over the gait cycle (Figure 2).

### Gait cycle parameters

The average stride interval time was significantly shorter during the high-heeled condition (Table 1). The variation in the stride interval time was not significant different between walking conditions (Table 1, “SD of stride interval time”).

**Table 1:** Stride interval time and standard deviation (SD) of stride interval time.

	Stride interval time (s)		SD of stride interval time (s)	
	Barefooted	High-heeled	Barefooted	High-heeled
Mean	1.07	1.01	0.012	0.015
SD	0.033	0.033	0.003	0.005
p-value	<0.00		0.08	

### Kinematics

**Figure 3:**
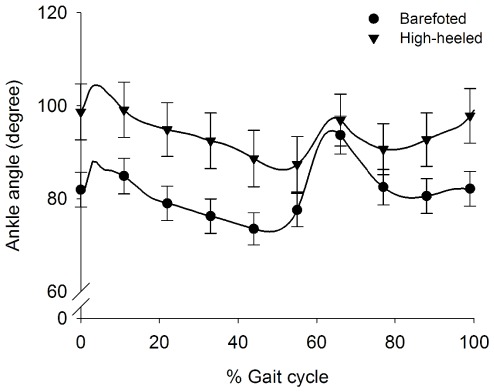
The ankle joint angle profiles. Ensemble average of the ankle joint angle (n = 11) during barefooted (dots) and high-heeled (triangles) walking. The ankle joint angle of each subject is a mean±SE of the ankle joint angle from 50 strides measured over 60 s of walking and used as input to the ensemble averaging. Each ankle joint angle signal was time normalized prior to averaging. The x-axis is expressed as % stride time where 0% and 100% identify heel strikes of the same leg.

The average ankle joint angle was significantly more plantar flexed in the high-heeled condition (15.1%) than in the barefooted condition (Figure 3). During barefooted walking the mean±SD ankle joint angle was 81.4°±12.2 compared to 93.7°±19.1 (p = 0.049) during high-heeled walking. Due to the shoe, the range of motion of the ankle joint was limited in the high-heeled condition compared to the barefooted condition (Figure 3).

**Table 2:** Approximate entropy (ApEn) of the joint ankle angle.

	ApEn	ApEn
Subject^a^	Barefooted	High-heeled
1	0.22	0.54
2	0.29	0.39
3	0.42	0.43
4	0.38	0.44
5	0.22	0.40
6	0.30	0.39
7	0.21	0.22
8	0.33	0.41
9	0.30	0.36
10	0.23	0.33
11	0.20	0.31
Mean^b^	0.28	0.38
SD	0.07	0.08
p-value	0.002	

a ApEn calculated over 60 s of barefooted and high-heeled walking for all subjects.

b The average ApEn (SD) of the whole group (n = 11).

### Movement variability

**Figure 4:**
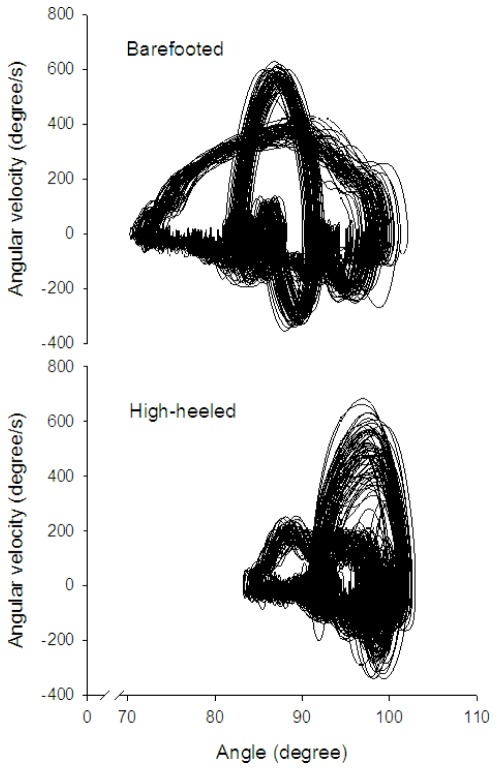
Representative phase portraits from one subject. Two-dimensional state spaces created by plotting the ankle joint position (x-axis; degrees) against the ankle joint angle velocity (y-axis; degrees/s). The ankle joint angle was collected over 60 s of barefooted (top panel) and high-heeled (bottom panel) walking. Note that the trajectories during high-heeled walking (bottom panel) display more divergence than during barefooted walking (top panel).

The variability of the angle joint angle signal was significantly increased during high-heeled walking (Table 2). The ApEn was 35.7% (p = 0.002) higher during high-heeled walking compared to barefooted walking and this was observed in all subjects (Table 2). This observation was further confirmed by visual inspection of the graphical representation of the phase portraits of the ankle joint angle (Figure 4). High-heeled walking was characterized by a higher degree of divergence in the trajectories while barefooted walking showed more regular and tight trajectories (Figure 4).

### H-reflex measurements

**Figure 5:**
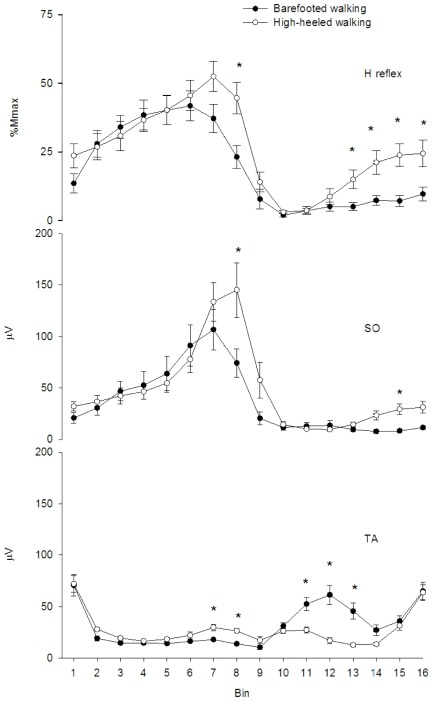
Mean SO H-reflex modulation and EMG activity. From the top: The mean SO H-reflex amplitude (% Mmax) and EMG activity (µV) of SO and TA during barefooted (filled circles) and high-heeled (open circles) walking for the whole group (n = 11). Gait cycle has been divided into 16 bins. Transition from stance to swing phase occurs at bin 10. Values are means±SE. Asterisks indicate statistically significant differences between the two walking conditions.

The absolute magnitude of the SO H-reflex measured in the standing position increased significantly from the barefooted (4.3±2.7 mV) to the high-heeled (7.3±2.8 mV) condition (p<0.001). Likewise the maximal M-wave (Mmax) increased significantly from the barefooted (9.0±3.7 mV) to the high-heeled (13.0±5.6) condition (p = 0.002). The SO H-reflex modulation showed significant differences between the high-heeled and the barefooted walking condition (Figure 5). The H-reflex modulation observed during barefooted walking resembled the classical pattern reported for normal human walking with a high amplitude during stance indicating facilitation and low amplitude during swing indicating inhibition. In contrast to this, the excitability of the H-reflex was in general increased in the high-heeled walking condition and this was most pronounced within the swing phase (Figure 5). This was observed in all subjects. Furthermore, during high-heeled walking the modulation pattern was characterized by a gradually increasing H-reflex excitability towards heel strike indicating facilitation within this phase (Figure 5). The mean H-reflex amplitude during swing (bin 10–16) was significantly increased by 182% in the high-heeled condition (p<0.001) (Figure 5). In the terminal stance phase (bin 8) the H-reflex amplitude increased significantly by 92% from the barefooted to the high-heeled condition (p<0.001) (Figure 5).

### Muscle activity and coactivation

**Figure 6:**
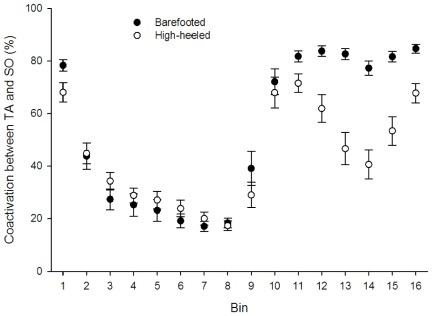
Level of coactivation between the SO and TA muscles. The coactivation was calculated as the relative contribution of the TA EMG to the total sum of the SO and TA EMG in each of the 16 bins representing the gait cycle. If the coactivation value approaches 50% the muscles contribute equally to the total amount of EMG activity indicating indicating maximal coactivation. 100% indicates that TA is active and SO silent while 0% indicates that the SO is active and the TA silent. Coactivation values of the high-heeled (open circles) and barefooted (filled circles) walking are means±SE.

The muscle activities of the SO and TA muscles were significantly changed in the high-heeled walking condition (Figure 5 and 6). The SO EMG activity was in general increased during high-heeled walking compared to barefooted walking (Figure 5). In the end of the stance phase (bin 8) the SO EMG activity was significantly increased by 95% from barefooted to high-heeled walking (p = 0.035) (Figure 5). The SO EMG activity increased gradually in the swing phase of high-heeled walking compared to barefooted walking where the muscle remained silent during the whole swing phase (Figure 5). The mean SO EMG activity in the swing phase was increased by 75.5% from the barefooted to the high-heeled condition (p = 0.028) (Figure 5). The EMG activity of the TA muscle was also significantly increased in the end of the stance phase during high-heeled walking (Figure 5). The TA EMG amplitude increased on average by 73% from barefooted to high-heeled walking (p = 0.025), (Figure 5). In contrast, the TA muscle activity was significantly reduced during the swing phase. The mean TA activity in the swing phase (bin 10–16) decreased by 39.7% from barefooted to high-heeled walking (p = 0.002), (Figure 5). However, the TA activity was only reduced in the middle of the swing phase in the high-heeled condition. In the high-heeled condition, the TA activity level was identical to that of barefooted walking in the terminal swing phase (bin 15–16, Figure 5). The changed muscle activity patterns observed in the high-heeled walking condition resulted in an increased level of coactivation between the SO and TA muscle (Figure 6). During high-heeled walking, the relative contribution of the TA EMG activity to the total EMG activity (i.e. the sum of TA and SO activity) approached 50% within the swing phase indicating increased coactivation between the two muscles (Figure 6).

## Discussion

We show that the ankle joint movement behavior of high-heeled walking was characterized by a more complex and variable pattern than barefooted walking. This was true for all subjects even though they were young, healthy and accustomed to high-heeled walking. The variation in the stride time intervals did not reveal increased variability during high-heeled walking. This finding supports other studies that claims that quantification of movement variability by simple descriptive statistics is insufficient because it does not measure the structure of the variability in the movement pattern [24]. Variability in human and animal movement is a common and well described phenomenon [16], [18], [32], [33], [34] and this variability has been interpreted differently within different theories of motor control [16], [35]. In some approaches movement variability has been considered as errors or undesirable noise in the motor programming [33] or as a result of redundancy in the motor control system [36]. However, others believe that movement variability has a functional role in human movement [16], [34]. Recently, Stergiou et al. (2006), proposed that movement variability has a deterministic structure ranging from pure periodic (totally predictable) over chaotic (complex and less predictable) to totally random (no predictability) which reflects the systems adaptability to the environment and external stimuli [16]. The relatively low ApEn values observed for both barefooted (0.28±0.07) and high-heeled (0.38±0.08) walking observed in our study illustrate the cyclic nature of walking and concur with ApEn results reported in previous studies of human walking [37]. Lower ApEn values have been reported for subjects with anterior cruciate ligament deficiency (ACL) when compared to healthy gait, meaning that the movement pattern of this patient group was more regular and less complex than the healthy walking pattern [38]. Furthermore, complexity in the walking pattern has been observed to increase during childhood and decline with aging and also in cases of neurological diseases [37]. Thus, the increased complexity and variability observed in the movement pattern of high-heeled walking may reflect the healthy motor control system's ability to cope with this unstable condition. All the participants in our study were young (range 20–38 years) and accustomed high-heeled walkers. Thus, the present results are only representative for young, healthy and skilled motor behavior of high-heeled walking and it is unknown how elderly and/or subjects with neuromuscular deficits would respond to an unstable walking condition as high-heeled walking. In addition to this it is unknown how young and healthy subjects who are unskilled in high-heeled walking would respond. Further studies are needed to reveal this.

In general, high-heeled walking has been reported to be characterized by increased plantar flexion and decreased ankle joint ROM [4], [6], [11], [39] as well as increased muscle activity of the SO and TA [4], [11], [12]. Our results are in agreement with these previous findings. Increased coactivation between the ankle joint muscles, due to higher SO EMG activity, was observed during high-heeled walking in the last part of the swing phase (Figure 6). This is in agreement with previous studies of high-heeled walking [4], [5], [6]. Increased coactivation between the ankle joint dorsal and plantar flexors will increase the joint stiffness [40], which presumably benefits the balance control around heel strike. This altered muscle activation pattern likely originates from changes in the central commands that shape the walking pattern. It is important to stress that the TA and SO muscle lengths were changed in the high-heeled condition, which may have influenced the EMG results. However, changes in the muscle length of the TA and SO muscles have been reported to have a limited influence on the EMG measurements [41]. A small decrease in the SO EMG at very short muscle lengths has been observed [41] but since we observed an increase in the EMG during high-heeled walking (i.e. shorter SO muscle length), we do not expect that the changed muscle length between the two walking conditions has biased the results.

Finley et al. [25] have recently shown that increased instability of the ankle joint increases the coactivation between the ankle plantar and dorsi flexor muscles. In the same study the stretch reflex amplitude was observed to be attenuated in the unstable situation suggesting that the control of an unstable ankle joint condition rely more on feed forward mechanisms (i.e. increased muscle coactivation) than on sensory feedback from the muscle spindles [25]. Our EMG results concur with these findings.

The exact mechanisms underlying the observed SO H-reflex modulation are unclear. It is possible that the combination of reduced TA EMG activity (i.e. reduced antagonist inhibition) in mid swing and a higher level of SO EMG activity in late swing (i.e. increased excitability of the SO motoneurones) is responsible for the increased SO H-reflex during high-heeled walking [27] (c.f. late swing, Figure 5). A centrally mediated reduction of the inhibition of the spinal monosynaptic reflex pathway may also have contributed to an increase in the SO H-reflex. The observed increased H-reflex in terminal stance of high-heeled walking is most likely due to the increased SO EMG activity also observed in this phase (c.f. late stance, Figure 5). However, the current data renders any conclusion about the mechanisms responsible for the observed H-reflex modulations tentative.

It is difficult to determine how the movement variability occurs. The changed muscle activation pattern and SO H-reflex modulation indicate that high-heeled walking is controlled differently from barefooted walking. It could be speculated that the increased SO H-reflex observed during late swing of high-heeled walking reflects that the motor control system is more open to sensory feedback. It has been suggested that the nervous system are capable of integrating feedback from many sensory modalities during unstable conditions to optimize balance control [25]. This will increase the number of degrees of freedom the system has to control, which could explain the increased movement variability in the high-heeled condition. However, existing evidence based on animal studies has shown that the movement variability may originate already in the preprocessing of the movement and therefore centrally generated [34]. On basis of our results we suggest that the control of the ankle joint movement during high-heeled walking was due to a changed control strategy that most likely increased the movement variability.

The current study focused on the control of ankle joint. It is well known that the contribution of the plantar flexors (especially the SO muscle due its huge PCSA) to overall gait performance (i.e. support and forward progression) is superior to the contribution of all other leg muscles [42]. However, many other joints, muscles and kinematic variables (i.e. centre of mass, knee joint) are relevant to investigate to fully understand how the nervous system controls high-heeled walking, which encourages further examination within this field.

In conclusion, the present results confirmed our initial hypothesis stating that the movement variability of the ankle joint angle would be increased significantly during high-heeled walking. Increased coactivation about the ankle joint together with increased excitability of the SO H-reflex in terminal swing phase was observed during high-heeled walking, indicating changes in the motor strategy of high-heeled walking. Thus, high-heeled walking needs to be controlled differently from barefooted walking in a way that is characterized by increased movement variability. We suggest that the higher variability reflects an adjusted neural strategy of the nervous system to control high-heeled walking.

## Materials and Methods

### Subjects

Eleven female subjects (mean (SD): age: 27.5 (5.4) years, height: 1.70 (0.04) m, body mass: 58.1 (5.1) kg) participated in the study. All subjects were accustomed to high-heeled walking. The subjects were exposed to two different walking conditions while they walked at 4.0 km/h on a motor driven treadmill (HS-1200 TechnoGym). One condition was barefooted walking while the other was high-heeled walking (heel height 9 cm). Each of the conditions consisted of two separate walking trials; 1) 60 s of walking where EMG, stride time and kinematics were measured and 2) measurements of the SO H-reflex excitability over the gait cycle. Prior to testing, all subjects were informed about the experiments and gave their informed consent to the conditions of the experiments, which were approved by the ethics committee for the Capitol Region of Denmark (in Danish: “De Videnskabsetiske Komiteer for Region Hovedstaden”) [j.no. H-4-2010-106] and experimental procedures were performed in accordance with the Declaration of Helsinki.

### Stride time and ankle joint angle

Two electrical microswitches were placed under the heel and forefoot of the left shoe or foot of each subject. The microswitches were used to identify the heel strike events and on basis of this the duration of the gait cycle (stride time) could be calculated. The ankle joint position was recorded by electrogoniometry (Penny & Giles, M-series). The signals were sampled at a frequency of 1000 Hz.

### EMG

Bipolar surface EMG electrodes (2DT2 Foam Dual Pregelled Electrode, Multi BioSensors Inc., USA) were placed over the prominent part of the SO and TA muscles according to the recommendations of Perotto [43]. The recording zone of each electrode had a diameter of 1.0 cm and the inter-electrode distance was 2.0 cm. The skin was carefully shaved and rinsed with pure alcohol, and the electrodes were connected to custom-built preamplifiers (input impedance, 80 MΩ) that were taped to the skin. A reference electrode was placed over the tibial bone. The EMG signals were led through long shielded wires to custom-built amplifiers with a frequency response between 20 and 10,000 Hz. The EMG signals from 60 s of walking were sampled at a frequency of 1000 Hz as the power spectrum contained no energy of significance above 500 Hz.

### H-reflex

The SO H-reflex was elicited by stimulating the tibial nerve every two seconds. A hand-held electrode was used to locate the optimum site of nerve stimulation, which was defined as exclusive stimulation of the SO Ia afferents. Then an AgCl cathode was placed on the skin in the popliteal fossa (Ambu VL-00-A) and a 10 cm^2^ anode was placed over the patella. The stimulus was a 1-ms square pulse delivered by a custom-built constant current stimulator and bipolar surface EMG electrodes on the SO muscle (2DT2 Foam Dual Pregelled Electrode, Multi BioSensors Inc., USA) recorded the M-wave and the SO H-reflex. Maximal H-reflex (Hmax) and M-wave (Mmax) were measured with the subjects in standing position in both conditions (barefooted/high-heeled). During walking the stimulation procedure was controlled by a computer program written in MATLAB. The stimuli given every 2 s corresponded to 25% (±10%) of the resting Mmax and were slightly out of phase with the gait cycle, which ensured the stimuli to be dispersed randomly over the gait cycle. The microswitch placed under the subject's heel reset an integrator at heel strike and a ramp function was generated from 0 to 2 V over a 2-s period (Figure 1). Knowing the slope of the ramp made it possible for the computer program to measure the temporal position in the gait cycle before applying the stimulus. The gait cycle was binned into 16 equal time slices and the H-reflex amplitude was averaged within each bin [44]. A minimum of four but typically ten H-reflexes were averaged within each bin and expressed relative to Mmax measured in the standing position. The H-reflexes and M-waves were sampled at a frequency of 10 kHz.

### Data treatment and calculations

The EMG and goniometer signals recorded over 60 s of treadmill walking were used for further analysis. The EMG signals were digitally high- and low-pass filtered (Butterworth fourth-order zero-lag digital filter, cut-off frequencies 20 Hz and 500 Hz, respectively), full-wave rectified and low-pass filtered at 15 Hz to produce linear envelopes. The signal from the microswitch on the heel was used to identify the heel strike of 50 gait cycles. Each gait cycle was time normalized by interpolation in MATLAB and subsequently averaged and divided into 16 bins to represent the EMG activity of the TA and SO muscles during the gait cycle. The EMG activity of both the SO and TA muscle was expressed in absolute values (µV). In addition, the coactivation between the SO and TA muscles was expressed as the relative contribution of the TA EMG activity to the total EMG activity (SO+TA) in each bin over the gait cycle, meaning that if each of the muscles were equally active the relative contribution of the muscles would be 50%.

The goniometer signal was low-pass filtered by a fourth order zero-lag Butterworth filter with a cut-off frequency of 50 Hz subsequently converted from volts to degrees and averaged over 50 gait cycles to determine the ankle joint position during the two walking conditions. The mean ankle joint position for each condition was then calculated. Each of the 50 gait cycles recorded in each subject was time normalized by interpolation in MATLAB to form curves of an equal number of data points that represented the gait cycle. Ensemble averages were then calculated for the high-heeled and barefooted condition using the mean ankle joint angle curve for each individual subject.

The movement variability of the ankle joint angle was assessed by quantifying the approximate entropy (ApEn) in the angle joint signal obtained over 60 s of walking [21]. The ApEn provides a single value that quantifies the regularity or predictability of a time series [21]. Low ApEn values indicate a more regular or periodic behavior, while higher ApEn values indicate more complex and unpredictable structures of the time series [16], [24]. The calculation of ApEn was done according to the method presented by Pincus, (1991). A brief explanation of this procedure is provided in the appendix in [24].

Phase portraits of the ankle joint angle were performed by plots of the ankle joint angle (x-axis) versus the ankle joint angle velocity (y-axis) (Figure 4) and used as a graphical representation of the degree of tightness/divergence in the trajectories in the high-heeled and barefooted walking conditions.

### Statistics

Student's t test for paired samples was used to determine if the ApEn of the ankle joint angle, stride time interval parameters, SO H-reflex amplitude, EMG amplitudes and the coactivation between SO and TA were significantly different between the barefooted and high-heeled walking condition. The comparisons of the H-reflex modulations and EMG activities were tested for significance at each of the 16 bins and the Bonferroni correction was applied by multiplying the p-values with the number of test (i.e. 16). The overall level of significance was set at p<0.05.
